# Why Is Murat’s Achievement So Low? Causal Attributions and Implicit Attitudes Toward Ethnic Minority Students Predict Preservice Teachers’ Judgments About Achievement

**DOI:** 10.3389/fpsyg.2022.819793

**Published:** 2022-03-31

**Authors:** Sabine Glock, Anna Shevchuk, Hannah Kleen

**Affiliations:** ^1^Institute for Educational Research in the School of Education, Bergische Universität Wuppertal, Wuppertal, Germany; ^2^DIPF | Leibniz-Institut für Bildungsforschung und Bildungsinformation, Frankfurt am Main, Germany

**Keywords:** causal attribution, implicit attitudes, ethnic minority students, ethnic bias, teacher judgment

## Abstract

In many educational systems, ethnic minority students score lower in their academic achievement, and consequently, teachers develop low expectations regarding this student group. Relatedly, teachers’ implicit attitudes, explicit expectations, and causal attributions also differ between ethnic minority and ethnic majority students—all in a disadvantageous way for ethnic minority students. However, what is not known so far, is how attitudes and causal attributions contribute together to teachers’ judgments. In the current study, we explored how implicit attitudes and causal attributions contribute to preservice teachers’ judgments of the low educational success of an ethnic minority student. Results showed that both implicit attitudes and causal attributions predicted language proficiency and intelligence judgments. Negative implicit attitudes, assessed with the IRAP, and internal stable causal attributions led to lower judgments of language proficiency, whereas lower judgments of intelligence were predicted by positive implicit attitudes and higher judgments of intelligence by external stable attributions. Substantial differences in the prediction of judgments could be found between the IRAP and BIAT as measures of implicit attitudes.

## Introduction

Schools around the world are becoming more culturally diverse, which includes students’ diversity not only in terms of cultural norms but also in terms of academic achievement and languages spoken in the classroom. Teachers are required to handle these diverse students and adapt their teaching as well as their classroom management strategies in order to successfully include ethnic minority students (Civitillo and Juang, 2020). However, ethnic minority students generally perform lower in academic achievement than their ethnic majority classmates (Musu-Gillette et al., 2016), and teachers tend to have lower expectations of ethnic minority students’ performance (Wang et al., 2018) even when students’ actual academic achievement is controlled for (Tenenbaum and Ruck, 2007). This judgmental bias concerning ethnic minority students also holds in Germany (e.g., Tobisch and Dresel, 2017; Bonefeld and Dickhäuser, 2018), where the largest ethnic minority group has Turkish roots (Statistisches Bundesamt, 2021). Besides teachers’ low expectations, their attitudes are potentially another major factor of this ethnic bias (Costa et al., 2021). Attitudes as evaluations of a social group (Eagly and Chaiken, 1993) can be divided into *implicit attitudes*, which occur automatically and usually outside of awareness, and *explicit attitudes*, which require conscious reasoning (Gawronski and Bodenhausen, 2006). The vast majority of studies have shown that teachers hold negative implicit attitudes toward ethnic minority students (Pit-ten Cate and Glock, 2019; Costa et al., 2021). In addition, expectations and evaluations are shaped by teachers’ causal attributions—an individual’s explanations for success or failure (Weiner, 2000)—for students’ outcomes (Reyna, 2008). Overall, research has shown that teachers are more likely to make internal and stable judgments about students’ failures, such as low ability, but teachers are significantly influenced by student ethnicity and tend to make more external attributions, such as luck for ethnic minority students’ academic success (Wang and Hall, 2018). However, it is not yet clear how these two factors—teachers’ implicit attitudes and causal attributions—affect teachers’ judgments in combination. To our knowledge, so far only one study by Glock and Kleen (2021) focused on this connection. Hence, the aim of this study is to investigate how implicit attitudes and causal attributions contribute to preservice teachers’ judgments of an ethnic minority student. The novelties of this study are an altered and extended questionnaire to assess causal attributions to get a more thorough insight into preservice teachers’ causal attributions and above that, implicit attitudes were administered with three different indirect methods. Furthermore, and in contrast to the study by Glock and Kleen (2021), a student vignette was employed to evaluate possible biased judgments of those preservice teachers.

## Implicit Attitudes

Attitudes are defined as an evaluation of a social group (Eagly and Chaiken, 1993). People can develop attitudes directly through their own personal experiences (Rudman, 2004) or indirectly through observing other people’s attitudes or reports in the media (Dovidio et al., 2010). Attitudes can be differentiated into implicit and explicit ones. With implicit attitudes, the evaluation is spontaneous and automatic, whereas explicit attitudes require conscious reasoning (Gawronski and Bodenhausen, 2006). However, as implicit and explicit attitudes can be considered two separable constructs, their relationships have often been found to range from no correlation to high positive correlations (Hofmann et al., 2005). Non-significant or low correlations between implicit and explicit measures can be found when assessing teachers’ attitudes toward socially sensitive issues, such as attitudes toward ethnic minority students (Pit-ten Cate and Glock, 2019). Hence, (preservice) teachers’ explicit attitudes toward ethnic minority students primarily tend to be positive (Glock et al., 2020). However, as the focus of attitude assessment has shifted toward the examination of implicit evaluations (Sritharan and Gawronski, 2010), research from the educational field has indicated that teachers show more negative implicit attitudes toward ethnic minority students (Glock et al., 2020). The same results have been found for preservice teachers, who tend to exhibit less favorable implicit attitudes toward ethnic minority students than toward ethnic majority students (Costa et al., 2021). Explicit attitudes are often assessed with self-report measures (Sritharan and Gawronski, 2010); implicit attitudes, however, are calculated from response latencies on tasks involving indirect measurement methods (Wittenbrink and Schwarz, 2007). As participants are required to evaluate stimuli as quickly as possible on such measures, deliberative processing is prevented (Denessen et al., 2020). Thus, implicit measures are less confounded with social desirability and can give deeper insights into the relationships between attitudes and students’ ethnicity.

The most prominent method for accessing implicit attitudes is the *Implicit Association Test* (IAT; Greenwald et al., 1998), which is used to calculate participants’ reaction times as they make cognitive associations. When applied to teachers’ implicit attitudes, it is assumed that teachers are quicker to assign positive attributes to ethnic majority students and negative attributes to ethnic minority students, the more strongly these concepts are cognitively linked (Costa et al., 2021). Hence, if the targets and attributes are strongly linked, the response latencies are shorter (Greenwald et al., 1998). The IAT has acceptable reliability (Hofmann et al., 2005) and validity (Greenwald et al., 2009) and has often been found to be superior to other implicit measures (Teige et al., 2004). However, despite its widespread use, the IAT has been criticized for producing measurement artifacts because it is assumed to be affected by non-associative influences, such as perceptual similarity, familiarity, or participants’ task-switching abilities (see Rothermund and Wentura, 2010; Johnson et al., 2021, for detailed overviews), resulting in substantial effects even when there is a lack of cognitive association between two concepts (Rothermund and Wentura, 2010). The same fundamental problems (Rothermund and Wentura, 2010) also affect the *Brief Implicit Association Test* (BIAT; Sriram and Greenwald, 2009), which is a shortened version of the IAT but is also quite different from the original IAT. In the original version of the IAT, all four combinations of targets and attributes are explicitly combined together (i.e., ethnic minority students and pleasant; ethnic minority students and unpleasant; ethnic majority students and pleasant; and ethnic majority students and unpleasant). By contrast, on the BIAT, only two of the combinations are explicitly combined, and the corresponding items are categorized with one computer key. The other two combinations are not explicitly mentioned, and the items in these categories are sorted using the other computer key. The greatest difference is that the focal categories are displayed on the screen, whereas the other categories are not displayed at all. The reliability and validity of the BIAT are acceptable (Sriram and Greenwald, 2009; Nosek et al., 2014).

Another promising measurement method, which also relies on the association between two concepts, but considers the extent to which two concepts are related, is the *Implicit Relational Assessment Procedure* (Barnes-Holmes et al., 2006). In the IRAP, participants are required to make congruent (e.g., ethnic minority students and good: different; ethnic minority students and bad: same) or incongruent (ethnic minority students and good: same; ethnic minority students and bad: different) responses. Results on the reliability and validity of the IRAP range from poor to moderate (Drake et al., 2015; Meissner et al., 2019; Sereno et al., 2021), but this measure is still in its infancy. Nonetheless, whereas the IAT and BIAT can access only relative attitudes (e.g., attitudes toward ethnic minority students relative to ethnic majority students), the IRAP is supposed to measure a person’s absolute attitude toward a target (e.g., an ethnic minority student) as positive, negative, or neutral (O’Shea et al., 2016). Given that the IAT is usually used in the educational context, and to exclude artifacts due to the method, we employed all three implicit attitude measures—the IAT, BIAT, and IRAP—to investigate the combined effects of implicit attitudes and causal attributions on judgments.

## Causal Attributions in the Educational Context

Causal attributions provide teachers with explanations about students’ academic achievements (Reyna, 2000), and teachers’ reactions are determined by what they attribute their students’ successes or failures to (Reyna, 2008). According to Weiner’s attributional theory, these causes can be internal or external as well as variable or stable (Weiner, 2000). For a student with successful academic achievement, an internal attribution may involve high abilities as a stable cause or effort as a variable cause (Weiner, 2000), whereas an external attribution may involve a teacher’s own educational practices as a stable explanation or luck as a variable explanation (H. Wang and Hall, 2018). The attribution can differ for success and failure and can be located either in (1) internal/stable aspects, such as low or high intelligence, (2) internal/variable aspects, such as high or low effort, (3) external/stable aspects, such as students’ parental support, or (4) external/variable aspects, such as task easiness or difficulty. Overall, teachers primarily attribute student failure to factors that are internal to the students, such as a lack of attention, motivation, or effort, and seldom to external/variable aspects concerning lesson difficulty or instructional quality (Wang and Hall, 2018).

Teachers’ attributions differ depending on ascriptive aspects of the students, such as students’ ethnicity (Reyna, 2000, 2008; Wang and Hall, 2018). In sum, studies have provided evidence that teachers tend to attribute academic success more to internal/variable factors (e.g., effort) for ethnic minority students in comparison to ethnic majority students. Teachers also tend to take external factors (e.g., their own teaching practices) into consideration to a greater extent when making attributions about ethnic minority students’ successful performances (Wang and Hall, 2018). But when ethnic minority students perform poorly, teachers tend to attribute failure primarily to internal, mostly stable causes and often neglect external aspects (Reyna, 2008). Such causal attributions may also affect teachers’ judgments of students’ performance. Likewise, if the failure of an ethnic minority student is attributed primarily to internal stable factors, the students’ competence is judged less favorably by preservice teachers (Glock and Kleen, 2021).

Another important aspect seems to be teachers’ assignment of high or low ability to a student (Wang and Hall, 2018). When teachers believe that a student has high ability, a failure is more likely to be attributed to external or unstable factors, such as task difficulty or low effort, whereas the failure of a low ability student would be attributed to low aptitude and thereby to internal stable factors (Reyna, 2000). Although attributions to unstable and therefore fluctuating causes should be more motivating for the students because they leave room for improvement in the future, teachers rarely attribute students’ failures to difficulty with lessons or poor instructional quality (Wang and Hall, 2018). Thereby, teachers’ attributions for student performance tend to be in line with teachers’ pre-existing expectations about students’ ability levels (Reyna, 2000), resulting in different attributions for students who are labeled high versus low achievers (Wang and Hall, 2018). The findings that ethnic minority students often perform poorly on academic tasks and are usually believed to have low ability (Reyna, 2000) reflect the fundamental attribution error (Ross, 1977) regarding ethnic minority students because teachers overestimate the role of dispositional factors and underestimate situational aspects when dealing with low performance (Wang and Hall, 2018). Indeed, previous studies have shown that teachers tend to rely on expectation-confirming information when judging ethnic minority students; that is, judgments about ethnic minority students were negatively biased when these students showed the low performance levels that were expected of them (Glock and Krolak-Schwerdt, 2013; Glock, 2016). This fundamental attribution error can have far-reaching effects on subsequent teachers’ classroom behavior, such as either encouragement or criticism directed toward such students (Wang and Hall, 2018). Taken together, teachers’ causal attributions reveal their beliefs regarding the reasons for their students’ academic achievement in terms of success and failure and can influence teachers’ expectations. When teachers make judgments about the low performance of ethnic minority students, a locus of causality on internal aspects seems to be predominant. The stability dimension plays an especially important role in expectations of future behavior (Reyna, 2008) and may therefore serve as a critical aspect for teachers, for example, when they make recommendations about which school track is appropriate for a student.

Causal attributions, implicit attitudes, and judgments can be brought together in the two-stage model of dispositional attributions (Trope, 1986), which assumes judgments as being based on an automatic process and on situational categorization. For the automatic process, the person’s group membership (Gawronski and Creighton, 2013) is relevant, which is also related to attitudes. In particular, implicit attitudes are activated by a person’s group membership (Fazio, 2001). During situational categorization, both internal and external causal attributions come into play, as people often infer internal causes of behavior (Uleman et al., 1996) and also consider situational constraints (Trope, 1986). Based on this model, teachers’ implicit attitudes toward ethnic minority students as well as their causal attributions regarding ethnic minority students’ achievements may play an important role in ethnic minority students’ disadvantages in school. Concurrent with this assumption, research showed that preservice teachers with more negative attitudes toward ethnic minority students and a tendency to attribute academic failure to internal stable factors made less favorable judgments about ethnic minority students’ competence (Glock and Kleen, 2021).

Accordingly, in the present study, we assessed teachers’ implicit attitudes toward ethnic minority students with three different measures, and we directly asked teachers to reflect on a particular scenario about a student’s failure. We assessed their causal attributions for the student’s low academic performance in the different main school subjects mathematics and German language proficiency as well as the student’s failure to get recommended for the highest school track. We expected that teachers with more negative implicit attitudes and more internal attributions of the student’s failures would judge the student as lower in academic achievement and lower in intelligence.

## Materials and Methods

### Participants

The 73 participating preservice teachers (58 women) were all in the master of education program at the university. On average, they were 24.74 (SD = 3.85) years old and had been teaching for an average of 30.22 weeks (SD = 46.02). Most of the preservice teachers (41.10%) focused on primary school. The focus of the remaining preservice teachers was unevenly distributed across the different secondary school tracks (30.13% focused on the highest school track; 13.70% on the lower secondary school tracks; 10.96% on vocational school). We had no information about participants’ ethnic minority background.

### Materials

#### Implicit Attitudes Tests

For each of the implicit tests, we implemented the same stimuli. We used six names, which indicated no ethnic minority background (Niklas, Leonie, Tim, Jonas, Emma, Marie), and six names, which implied an ethnic minority background (Cem, Erkan, Gökhan, Salim, and Elif, Filiz). We also employed six positive (lovingly, warm, fair, honest, funny, and helpful) and six negative adjectives (harsh, toxic, lying, ruthless, two-faced, venally).

#### Causal Attributions

We compiled a questionnaire on causal attributions for the missing educational success of ethnic minority students. We used the classical four dimensions as proposed by Weiner (2000) and additionally separated the causal attributions for students’ low achievement in Mathematics, German, and for their failure to be recommended for the highest school track (see Table 1 for all items of the different dimensions and Cronbach’s alpha for the subscales).

**Table 1:** Items of the causal attributions questionnaire and Cronbach’s alphas for the four Weiner’s dimensions.

Dimension	Items	Cronbach’s alpha
**Internal stable attributions**		0.83
	*Murat is achieving low in the Mathematics because…*	
	…he has low intellectual abilities	
	…he has a low numerical understanding	
	…he has a low ability for spatial reasoning	
	*Murat is achieving low in German because…*	
	…he has low intellectual abilities	
	…his vocabulary is limited	
	…he has a low knowledge of the grammar	
	*Murat is not receiving a recommendation for the highest school track, because…*	
	…he has low intellectual abilities	
	…he hast low language proficiency	
	…he has low mathematical aptitude	
**Internal variable attributions**		0.84
	*Murat is achieving low in Mathematics because*…	
	…he does not invest much effort	
	…he has not learned enough	
	…he does not participate in the lessons	
	*Murat is achieving low in German because…*	
	…he does not invest much effort	
	…does not have enough practice in speaking German	
	…he avoids speaking German	
	*Murat is not receiving a recommendation for the highest school track because…*	
	…he does not invest much effort	
	…he is not motivated	
	…he does not work thoroughly enough	
**External stable attributions**		0.88
	*Murat is achieving low in Mathematics because…*	
	…his parents deem Mathematics as less important	
	…his parents are not able to help him with his Math problems	
	…his parents cannot effort additional learning materials	
	*Murat is achieving low in German because…*	
	…his parents are not very fluent in German	
	…his parents deem speaking German as not important	
	…his parents cannot effort books	
	*Murat is not receiving a recommendation for the highest school track because…*	
	…his parents are not familiar with the German school system	
	…his parents are not able to sufficiently support him	
	…his parents are familiar with the values of the different secondary school types	
**External variable attributions**		0.74
	*Murat is achieving low in Mathematics because…*	
	…he cannot well understand story problems in Mathematics	
	…if he can freely choose the tasks, he always chooses too difficult tasks	
	…he is often underestimated because of the teachers’ conceptual formulation	
	*Murat is achieving low in German because…*	
	…essays and dictations are difficult for him	
	…the task selection is too one-sided	
	…he is often underestimated because of the teachers’ conceptual formulation	
	*Murat is not receiving a recommendation for the highest school track because…*	
	…he cannot develop adequately due to the low task difficulty	
	…if he can freely choose the secondary school track, he chooses the lowest track	
	…the recommendation for the secondary school track does not mirror his actual achievement	

The means of the Weiner’ subscales were calculated using nine items, of which three referred to the achievement in mathematics, three to the achievement in German, and three to the failure of receiving a recommendation for the highest school track.

#### Student Vignette

We used the same student vignette as in previous research (Glock and Kleen, 2019). Within this vignette, a student named Murat is described as low achieving, not very motivated, and showing working and learning habits which are at a low level.

#### Judgment Dimensions

For the judgment of language proficiency, we averaged the mean across the judgments of grammar, orthography, language, and reading comprehension (Cronbach’s *α* = 0.89). The other judgment dimensions were intelligence and mathematics, which were both assessed with single items.

#### Demographic Questionnaire

We compiled a demographic questionnaire assessing participants’ age, gender, the school type they majored for, and their teaching experience in weeks.

### Procedure

**Figure 1:**
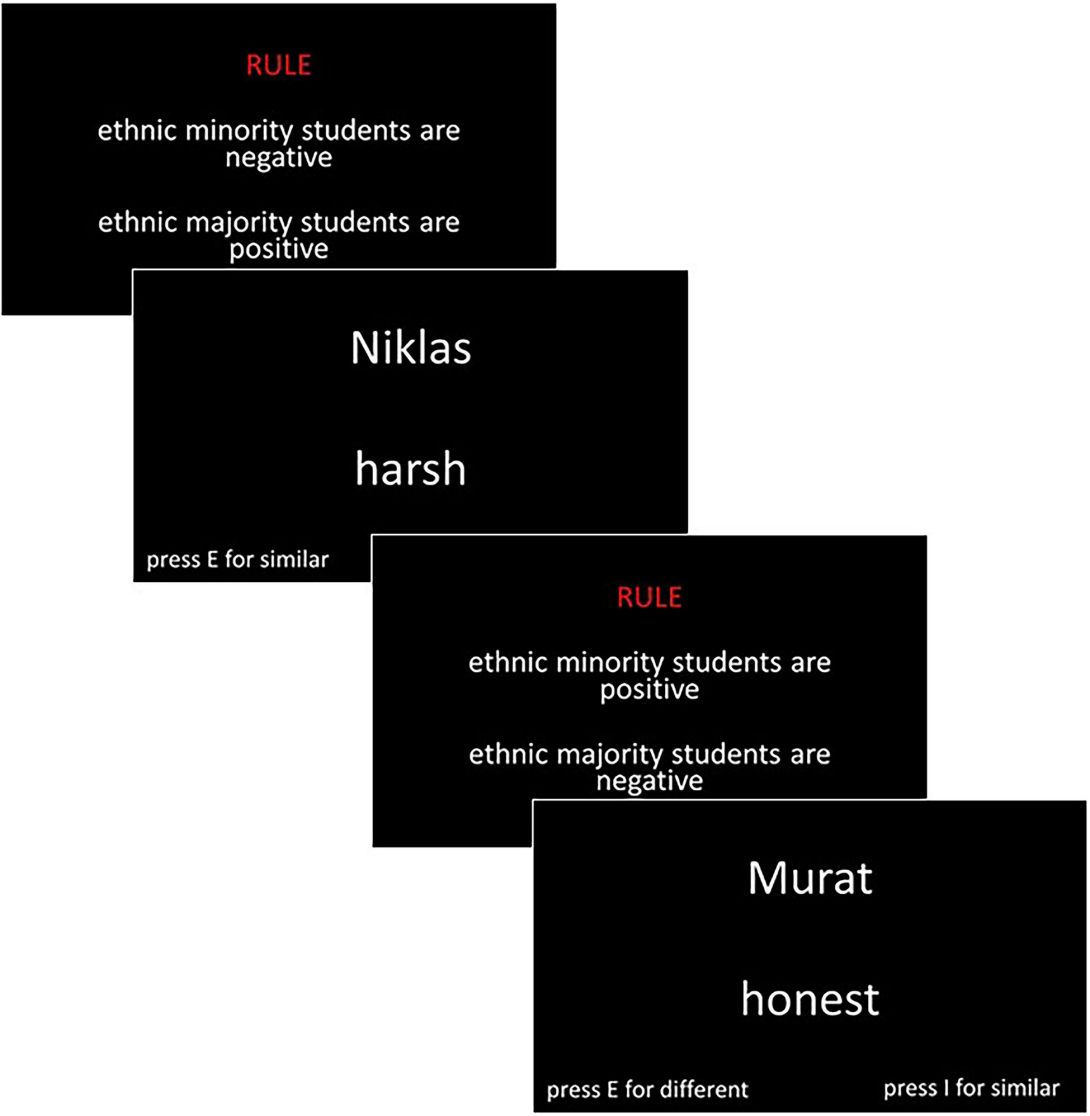
Schematic presentation of the IRAP.

The study was conducted as an online study. The link was distributed in the preservice teachers’ courses at the university and *via* personal contact. The participants first gave informed consent and were informed that the study was about how preservice teachers perceive students from ethnic minorities and ethnic majorities. Then, the three different implicit attitude tests were administered in a random order. In the following, we describe one of the original orders, beginning with the description of the IRAP. First, participants were instructed that they would be presented with positive and negative adjectives as well as with names that would indicate either an ethnic minority or an ethnic majority background. They were informed that their task was to decide whether the names and adjectives were similar in valence. The valence of the names was presented as a rule for each block and changed at random. Hence, there were three blocks in which the rule was that ethnic minority names were paired with negative adjectives and ethnic majority names were paired with positive adjectives. In these blocks, the participants were asked to press the “I” button for “similar” when ethnic majority names appeared with positive words and when ethnic minority names appeared with negative words. When the other two combinations appeared (i.e., when ethnic minority names were paired with positive words and ethnic majority names were paired with negative words), participants were asked to press the “E” key for “different.” In the three additional blocks, the rule was changed (please see Figure 1 for details).

Hence, in these blocks, ethnic minority names were paired with positive adjectives and ethnic majority names with negative ones. For each combination of the blocks, a practice block of 15 trials preceded the two test blocks, which consisted of 30 trials each. For each participant, the assignment of the keys and the ordering of the blocks was randomized.

**Figure 2:**
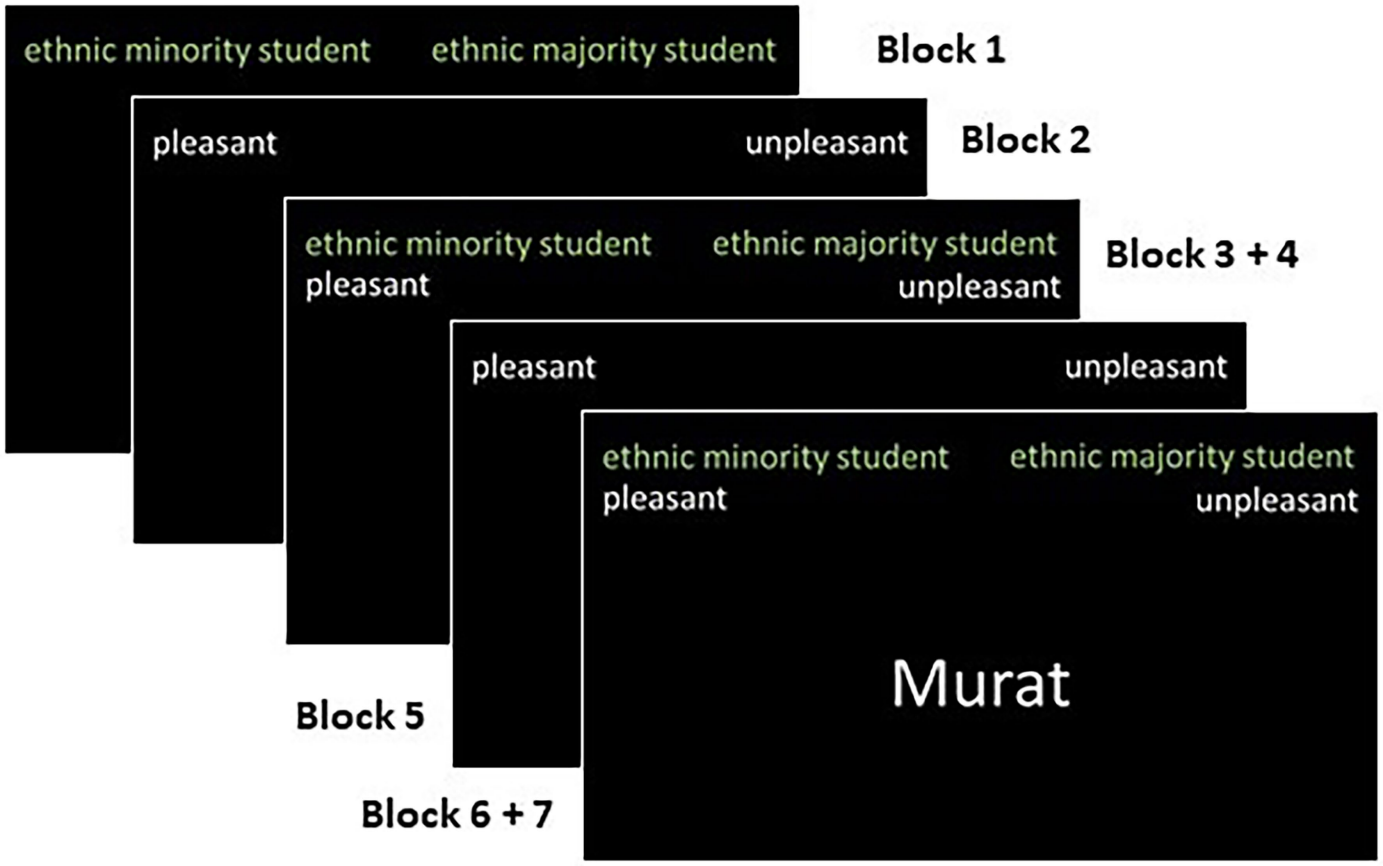
Schematic presentation of the IAT.

The next implicit test was the IAT. The IAT began with the categorization of the positive and negative words into the categories “pleasant” and “unpleasant” using the “E” and the “I” keys on the keyboard. After this, the participants were asked to use the same two keys to categorize the ethnic minority and majority names into the categories “ethnic minority student” and “ethnic majority student.” In the compatible combination of the two tasks, the participants sorted ethnic minority names and unpleasant words using the same key (e.g., the “E” key) and ethnic majority names and pleasant words using the other key. After this combination, the categories “ethnic minority students” and “ethnic majority students” switched sides of the computer screen along with the corresponding key. With this reversed and incompatible combination, participants now sorted ethnic minority students and positive words using one key and ethnic majority students and negative words using the other key (please see Figure 2 for details).

Overall, participants worked on 20 practice and 30 test trials for each combination. We shortened the numbers of trials in order to keep the time for the implicit tests to a minimum. For each participant, the assignment of the keys and the ordering of the compatible and incompatible blocks were randomized.

**Figure 3:**
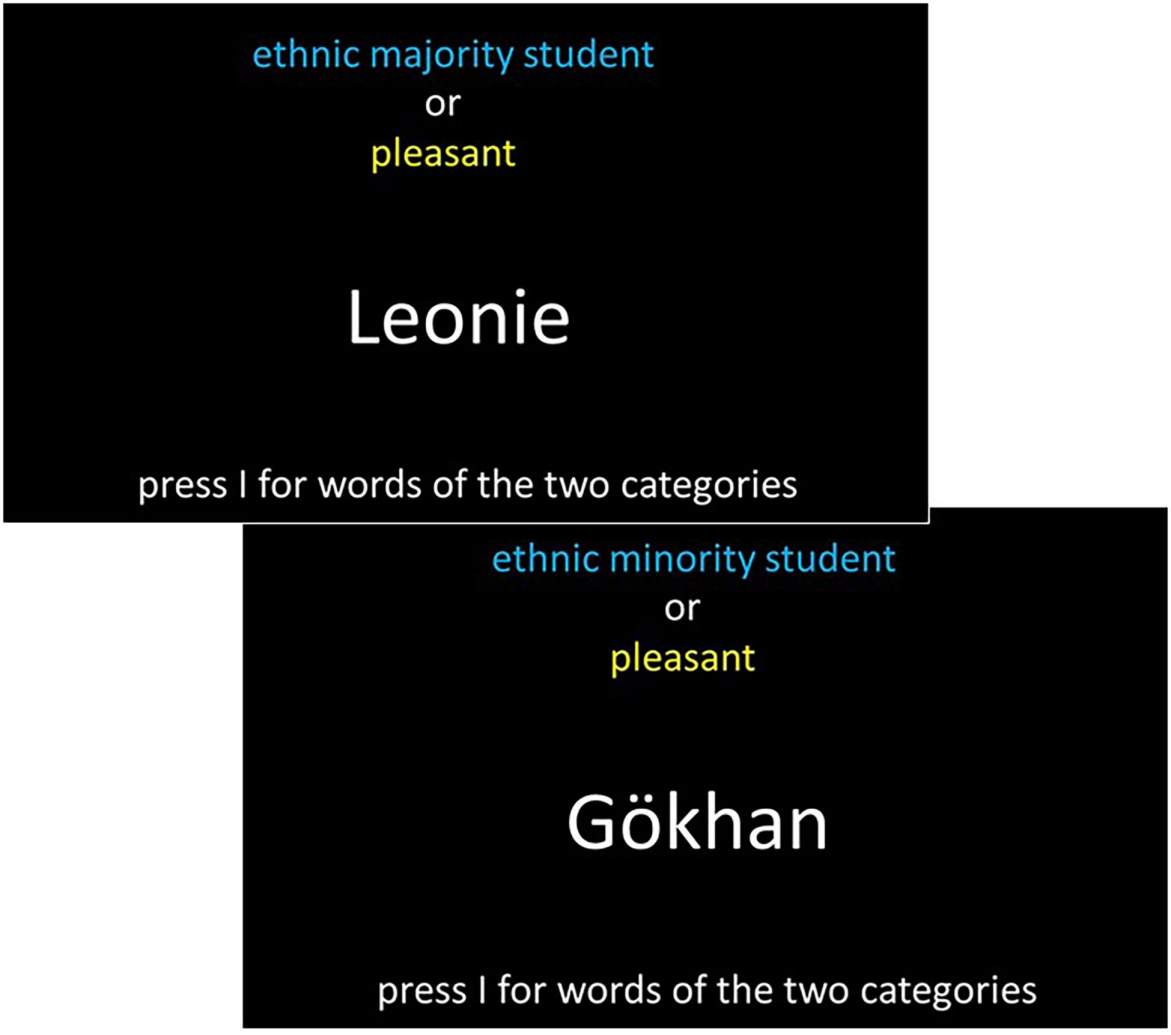
Schematic presentation of the BIAT.

As the last implicit attitude test, the BIAT was administered. This test used the same categories as the IAT, but in each block, there were focal categories. For instance, in compatible blocks, the focal categories were “ethnic minority students” and “unpleasant,” and the non-focal categories were “ethnic majority students” and “pleasant.” Hence, even though during instruction, only the focal categories were mentioned as a rule, the items from the non-focal categories also appeared and had to be sorted. In the compatible blocks, the focal categories also changed, so the categories “ethnic minority students” and “pleasant” were the focal categories, whereas the other two categories became non-focal. In the incompatible blocks, the pairing was switched so that “ethnic minority students” and “pleasant” were focal and “ethnic majority students” and “unpleasant” were non-focal. In these compatible blocks, the roles of the focal and non-focal categories were also switched. Participants used the “E” and the “I” keys to indicate the focal and non-focal categories. The assignment of the keys changed for each participant as well as the ordering of the blocks. The practice blocks consisted of 12 trials for each of the compatible and incompatible combinations and 40 test trials in each block combination (please see Figure 3 for details).

When the participants had finished all the implicit tests, the student vignette was presented. Participants were asked to read it carefully, and afterwards, they were asked to judge the student’s language proficiency, mathematics ability, and intelligence on a 7-point Likert scale ranging from 1 (*low*) to 7 (*high*). Following this, participants were given the causal attributions questionnaire. On a scale ranging from 1 (*fully disagree*) to 5 (*fully agree*), they indicated their agreement with the statements, which all related to the particular student they read about in the vignette. In the end, participants were thanked and debriefed.

## Results

### Statistics

For all the implicit tests, we calculated scores for the implicit attitudes according to the scoring algorithm proposed by Greenwald et al. (2003). First, extreme responses are excluded, that means responses that were longer than 10 s or shorter than 300 ms. If more than 10% of trials had to be deleted for one participant, this participant was excluded from further analysis. The error latency was replaced with the block mean of correct responses plus a penalty which was computed by the averaged standard deviation of correct responses added to the response time for the incorrect response. To obtain the *D-*score, the difference between the average response latencies between the two contrasted conditions, compatible and incompatible trials, was calculated including both the practice and the test trials. Those scores were then divided by their respective pooled standard deviations. The quotients for the two trials were averaged, representing the *D*-score, which indicates the strength and direction of participants’ implicit association. The *D*-score is interpreted such that positive values indicate more negative implicit attitudes toward ethnic minority students. For the implicit measures, it is possible to investigate the nature of the attitudes using a one-sample *t*-test, in which the *D*-score is tested against a score of 0, which indicates neutral attitudes. In the following, the *D*-score, one-sample *t*-test statistics, and Cohen’s d*s* effect sizes are reported.

### Descriptive Results

Because of the very low reliability of the IAT, we excluded this implicit measure from further analyses. For the reliability of the three tests (BIAT, IRAP, and IAT), we calculated the correlation of each test’s test and practice scores as recommended by Greenwald et al. (2003). The internal consistencies for the three measures were *r* = 0.79 for the BIAT, *r* = 0.70 for the IRAP, and *r* = 0.26 for the IAT. We first calculated the means and the standard deviations for all the dependent measures and computed the intercorrelations (see Table 2 for the correlations).

**Table 2:** Correlations between the different dependent variables.

S. No.	*Variable*	1	2	3	4	5	6	7	8	9
1.	Intelligence	1	0.23	0.41^*^	−0.21	−0.15	0.20	0.05	0.16	0.13
2.	Language		1	0.10	−0.43^*^	−0.11	0.08	−0.07	0.09	−0.26^*^
3.	Mathematics			1	0.03	−0.12	−0.28^*^	0.25^*^	0.16	0.11
4.	Internal stable				1	0.50^*^	0.34^*^	0.54^*^	−0.11	0.02
5.	Internal variable					1	0.22	0.19	−0.07	−0.01
6.	External stable						1	0.70^*^	0.00	−0.14
7.	External variable							1	−0.05	−0.11
8.	BIAT								1	−0.25^*^
9.	IRAP									1

BIAT, Brief Implicit Association Test; IRAP, Implicit Relational Assessment Procedure.

* *p* < 0.05.

Not surprisingly, students who received higher judgments of their mathematics ability from the preservice teachers were also rated as more intelligent. The more the participants endorsed internal stable attributions, the more they agreed with the remaining three attributional subscales. However, not each attributional dimension was correlated with the others. The two external subscales were correlated with each other but not with the internal variable dimension. Interestingly, the two implicit measures were negatively correlated, indicating that the more negative the implicit attitudes on the BIAT were, the more positive the implicit attitudes on the IRAP were.

For the BIAT, implicit attitudes were negative (*M* = 0.23, SD = 0.34), *t*(72) = 5.82, *p* < 0.001, *d* = 0.67, as they were for the IRAP (*M* = 0.09, SD = 0.34), *t*(71) = 2.31, *p* = 0.024, *d* = 0.26.

We computed one-sample *t*-tests for the causal attributions and compared the means with 3 as the middle of the scale. The participants made lower internal stable (*M* = 2.56, SD = 0.70), *t*(71) = 5.35, *p* < 0.001, *d* = 0.63, and external variable attributions (*M* = 2.52, SD = 0.80), *t*(71) = 5.03, *p* < 0.001, *d* = 0.60. They made higher internal variable attributions (*M* = 3.31, SD = 0.82), *t*(71) = 3.19, *p* = 0.002, *d* = 0.38. The external stable attributions were near the middle of the scale (*M* = 2.93, SD = 0.62), *t*(71) = 0.92, *p* = 0.36, *d* = 0.11.

### Multiple Regression Analysis

In order to predict the judgments with attitudes and causal attributions as suggested by the two-stage model of dispositional attributions (Trope, 1986), we conducted three different multiple regression analyses (see Table 3). Because of the relatively high intercorrelations of the predictors, we checked for multicollinearity, which was not a problem. All VIF values were below 10 as suggested by Field (2018).

**Table 3:** Summary of the multiple regression analyses with implicit attitudes, the four dimensions of causal attributions as predictors and the judgment dimensions as criteria.

Predictor	*B*	*95% CI for B*		*SE B*	*B*	*R* ^2^
		*LL*	*UL*			
*Language proficiency*						0.30
Internal stable	−0.83^*^	−1.24	−0.43	0.20	−0.60^*^	
Internal variable	0.15	−0.14	0.447	0.15	0.13	
External stable	0.24	−0.24	0.72	0.24	0.15	
External variable	0.12	−0.30	0.53	0.21	0.10	
BIAT	−0.09	−0.73	0.54	0.32	−0.03	
IRAP	−0.65^*^	−1.28	−0.13	0.32	−0.22^*^	
*Mathematical ability*						0.19
Internal stable	−0.04	−0.50	0–42	0.23	−0.03	
Internal variable	−0.21	−0.54	0.12	0.17	−0.17	
External stable	0.43	−0.11	0.97	0.27	0.26	
External variable	0.18	−0.29	0.64	0.23	0.14	
BIAT	0.59	−0.13	1.30	0.36	0.19	
IRAP	0.64	−0.07	1.36	0.36	0.21	
*Intelligence*						0.20
Internal stable	−0.36	−0.81	0.10	0.23	−0.24^*^	
Internal variable	−0.11	−0.44	0.22	0.17	−0.09	
External stable	0.61^*^	0.07	1.15	0.27^*^	0.37^*^	
External variable	−0.06	−0.52	0.40	0.23	−0.05	
BIAT	0.57	−0.14	1.23	0.36	0.19	
IRAP	0.69^+^	−0.03	1.40	0.36^+^	0.23^+^	

BIAT, Brief Implicit Association Test; IRAP, Implicit Relational Assessment Procedure.

* *p* < 0.05.

+ *p* = 0.05.

The judgments of German language proficiency were significantly predicted by internal stable causal attributions (*β* = −0.60, *p* < 0.05). More specifically, the more the preservice teachers thought that the ethnic minority student’s lower success in school was due to the student’s internal variable causes, the lower they judged the student’s language proficiency. The more negative the participants’ implicit attitudes toward ethnic minority students were when assessed with the IRAP, the lower the participants judged the German language proficiency of the student.

The judgments of intelligence were significantly predicted by external stable attributions (*β* = 0.37, *p* < 0.05). The more the preservice teachers indicated that they believed that the student’s failure in the educational system was due to external stable reasons (e.g., the parents), the higher they judged the student’s intelligence. Moreover, the more positive the preservice teachers’ implicit attitudes were when assessed with the IRAP, the lower they judged the student’s intelligence.

The judgments of mathematics ability were not predicted by any of the independent variables.

## Discussion

The results of this study demonstrate that preservice teachers’ implicit attitudes as well as their attributions of ethnic minority students’ low success in education play a role when judging an ethnic minority student’s scholastic achievements. However, this finding did not hold for all three of the judgment dimensions, and furthermore, only the IRAP, but not the BIAT, predicted preservice teachers’ judgments. This is especially interesting because the two measures, even though they are implicit, do differ, which could be important for future studies and might provide some first indications of (preservice) teachers’ judgment processes. The BIAT is relative in nature because, even though not every combination of categories is focal, the related stimuli still appear and still need to be sorted. This means that statements about one category (e.g., ethnic minority students) should always be viewed in relation to the other category, in this case ethnic majority students. For the IRAP, however, combinations were fixed, and participants were not required to discriminate between ethnic minority and ethnic majority students but were instead asked to react to the presented combinations. Comparing the IRAP and the BIAT in this study, participants sorted items on the BIAT, whereas they judged combinations as similar or different on the IRAP. Therefore, it has been suggested that the IRAP is a measure that is less relative than the BIAT or IAT and assesses beliefs instead of associations (Gawronski and De Houwer, 2014). Even though the lower relativity of the IRAP might not hold in this study because we also applied the scoring algorithm to the IRAP measures, the suggestion that it captures beliefs (Barnes-Holmes et al., 2006) might still have led to the different influences of the results of the two indirect measures. For example, evaluating ethnic minority students and negative adjectives as similar might even more precisely represent implicit beliefs about ethnic minority students than when participants only had to sort words, but no evaluation of similarity had to be given. This might be why the IRAP, but not the BIAT, predicted the judgments. In this respect, the IRAP could be a promising implicit method to use in future studies on (preservice) teachers’ implicit attitudes. However, these inferences are speculative and should be more deeply investigated in future research, particularly given the initial indications of only moderate reliability (Gawronski and De Houwer, 2014). Nevertheless, and despite the fact that the IAT could not be considered in the analyses, this is the first study (1) to compare the IRAP and the BIAT in relation to preservice teachers’ attitudes toward ethnic minority students and (2) to use these implicit measures as predictors of preservice teachers’ judgments of ethnic minority students’ achievements. However, more research is needed using more than one implicit measure to investigate how these are interrelated. Often, different implicit measures do not correlate (Payne et al., 2008; Glock and Karbach, 2015), which also shows that different measures tap into different automatic constructs.

Even though the ethnic minority student was performing low in both German language proficiency and mathematics, only the judgment of German language proficiency was predicted by preservice teachers’ causal attributions and implicit attitudes. Internal stable attributions of low success in school and more negative implicit attitudes led to lower language performance ratings. The internal attributions are in line with previous research (Froehlich et al., 2016; Glock and Kleen, 2021), but the separate consideration of internal variable and internal stable attributions is new and is especially interesting as previous research showed that ethnic minority students’ language problems were often attributed to low effort (Agirdag et al., 2014) and thus to internal variable attributions. In the current study, the preservice teachers attributed the ethnic minority student’s German language proficiency to the student’s ability. Such attributions can have disadvantageous consequences for ethnic minority students; for example, when teachers adjust their feedback to the students accordingly but fail to recognize that feedback is more beneficial for students when the teacher attributes a student’s achievement to internal variable reasons (Hattie and Timperley, 2007). One explanation for this result—especially because internal stable attributions predicted only German language judgments but not mathematics ability or intelligence judgments—could be that ethnic minority students are often viewed as having low levels of performance in German (e.g., Bonefeld and Dickhäuser, 2018; Kleen and Glock, 2018a) and low German language proficiency (Kahraman and Knoblich, 2000; Bonefeld and Karst, 2020). Furthermore, in past decades, German language proficiency has not increased as much for Turkish students as for other ethnic minority students (Müller and Stanat, 2006) and tends to be worse for Turkish students compared with other ethnic minority students from kindergarten to university (Olczyk et al., 2016). Hence, low language proficiency could be seen as a stable, especially for Turkish students.

In addition to the internal stable attribution, implicit attitudes predicted German language judgments in that more positive implicit attitudes predicted higher German language judgments. The correlation between German language judgments and implicit attitudes was not found in a previous study (Glock and Kleen, 2021). However, this previous study used the IAT as the implicit measure, which cannot easily be compared with the BIAT or the IRAP. Interestingly, we found a completely different pattern regarding the predictive value of implicit attitudes for the judgments of students’ intelligence. More positive attitudes, as measured with the IRAP, were related to lower intelligence ratings. Additionally, ethnic minority students’ intelligence was judged as higher when participants attributed school failure to external stable reasons, for example, to the parents. Even though the latter might be plausible, such a result for implicit attitudes was unexpected. Perhaps this finding was a result of justification processes (Crandall and Eshleman, 2003) in the sense that more positive implicit attitudes might lead to a lower influence of social norms, and participants might be more likely to reveal their actual opinions about the student’s intelligence. Especially because information about mathematical and language proficiency was included in the student vignette but no information was given about intelligence, participants’ intelligence judgments may have been based on inferences that went beyond the given information. Therefore, the influence of implicit attitudes might have been different because the amount of information can affect the nature of the relationships between attitudes and judgments (Brewer, 1996; Gawronski and Bodenhausen, 2006). However, this is highly speculative and needs to be further validated in the future.

Furthermore, neither internal/external attributions nor implicit attitudes predicted preservice teachers’ judgments of students’ mathematical ability. This finding is—with the exception of external attributions—contradictory to findings from previous research (Glock and Kleen, 2021). One explanation for the diverging results could be the different ways in which preservice teachers’ judgments were assessed. Whereas they were previously assessed with a semantic differential, a vignette describing the student was used in the current study. Hence, the additional information about the students’ mathematical abilities might have provided the participants with data to base their judgments on. More information about a person can hinder the influence of categorical thinking (Brewer, 1996) and might explain these results. That this was only true for the judgment of mathematical ability might reflect stereotypes about ethnic minority students. Mathematics is not as strongly associated with stereotypes of ethnic minority students as German language proficiency is (Bonefeld et al., 2021), hence leaving less room for the impact of attitudes and stereotypical causal attributions.

Our results also have educational implications. The awareness of these stereotypical causal attributions and a training to incorporate more external and particularly, variable rather than stable attribution styles might help teachers to overcome the assumptions, that low language proficiency is a stable factor for Turkish students. Experimental studies show, that people can be encouraged to see capacities, such as intelligence as modifiable instead of determined (Aronson et al., 2002) and attribute academic difficulties rather to external causes (Good et al., 2003). Even mental rotation as a facet of intelligence can be trained (Moè, 2016) and teacher motivation is one key element in the training of students (Moè and Katz, 2021). Therefore, language proficiency can be seen as a malleable ability, which can be changed by external factors, such as a more adaptive education style by teachers to Turkish students. Hence, when teachers provide different learning materials for Turkish and German students, the reason for insufficient language proficiency and therefore failure might no longer be seen as an internal stable variable. Teachers could be trained to believe that ethnic minority students can succeed and that their educational style has a higher impact than they expect it to be. The same training might be offered to the students as studies have shown that the awareness of negative stereotypes can influence the performance of students in a detrimental way, which is known as stereotype threat (Steele and Aronson, 1995). Thus, ethnic minority students might suffer in their performance when negative stereotypes about them as a group are salient.

## Limitations

In the analyses, the IAT had to be excluded due to low internal consistency. One reason for the low internal consistency could be the reduced number of trials. In comparison with other studies (e.g., Glock and Kovacs, 2013; Kleen and Glock, 2018b; Glock and Kleen, 2020) in which the reliability was higher, this IAT was shortened because participants were administered three different implicit measures. In future research, it might be beneficial to employ the complete IAT because a version with a smaller number of trials seems to reduce its reliability.

Another limiting aspect could be the choice of participants, as we only asked preservice teachers for participation. Even though a meta-analysis showed that there are no differences between preservice and in-service teachers when it comes to implicit attitudes (Pit-ten Cate and Glock, 2019), it would be of interest to investigate to which extent preservice and in-service teachers differ regarding their causal attributions. This is especially important, as teachers often see internal factors of students as responsible for those students’ scholastic failure instead of, for example, factors that teachers can influence (Wang and Hall, 2018). In future research, we might also differentiate between mathematics and German language as the main school subjects. This might be of particular interest as primary school teachers are required to teach both subjects, which would allow us to hold the teacher constant across the school subjects.

Furthermore, this study did not include judgments about German students. Future studies should include a vignette about a German student as a comparison. To our knowledge, (preservice) teachers’ judgments about ethnic minority versus ethnic majority students’ scholastic achievements have not yet been comparatively analyzed using teachers’ attributions in addition to their implicit attitudes. Another study employed a semantic differential with Turkish compared with German students as poles (Glock and Kleen, 2021), but this previous study did not use such a vignette like we did in the current study (or judgments based on a vignette). Particularly because judgments of Turkish versus German students have been shown to be different (Glock, 2016; Bonefeld and Dickhäuser, 2018; Kleen and Glock, 2018a), it would be of great interest to obtain more information about predictors of (preservice) teachers’ judgments. Relatedly, the socio-economic background of the student should also be investigated, as research has shown than students from families with low socio-economic status are also vulnerable to get stereotyped (Dunkake and Schuchart, 2015; Glock and Kleen, 2020).

In line with the lack of a vignette about a German student, (preservice) teachers’ attributions have also only been investigated for ethnic minority students in this kind of research to date. Future research should include ethnic majority students. As ethnic minority students are often a negatively stereotyped group (Reyna, 2008), attributions of educational success or failure can be different for ethnic minority versus ethnic majority students. Thus, such potential differences should be explored in more detail. So far, to our knowledge, no study has examined (preservice) teachers’ attributions of German students’ educational failures in contrast with Turkish students’ educational failures. Moreover, because research has shown differences in attributions of failure and success between high- and low-achieving students (Wang and Hall, 2018), it might also be of great interest to ask preservice teachers about their causal attributions for the high academic achievement of an ethnic minority student.

Last but not least, we only focused on attitudes toward and attributions as well as judgments of Turkish students. Future research could also focus on investigations of teachers’ implicit attitudes and especially their attributions of other ethnic minority students’ school failure and success. As attributions are associated with stereotypes and as stereotypes differ for various social and ethnic groups (Fiske et al., 2002; Asbrock, 2010), attributions for the successes and failures of different groups might also vary. One example would be Asian students who are a high-achieving ethnic minority group (Walter, 2011) and are often viewed as hard-working (Lin et al., 2005). Hence, Asian students’ success in school might be attributed to internal variable instead of internal stable attributions. Therefore, for instance, feedback could be more beneficial for this group and as a result may also contribute to their higher success in school because even though preservice teachers endorsed internal variable attributions more than they endorsed internal stable attributions for the Turkish students in the current study, the internal stable attributions predicted their judgments.

Despite these limitations, this is the first study to compare the IAT, BIAT, and IRAP as implicit measures and predictors of preservice teachers’ judgments and the second to additionally include preservice teachers’ attributions of ethnic minority students’ school failure. What is more, we categorized attributions into those that are internally stable and variable and those that are externally stable and variable and were therefore able to shed more light on teachers’ attributional associations and judgment processes.

## Data Availability Statement

The raw data supporting the conclusions of this article will be made available by the authors, without undue reservation.

## Author Contributions

SG: conceptualization, literature search, analyzing the data, writing the original draft, and revision and editing of the manuscript. AS: conceptualization, collecting the data, writing the original draft, and revision and editing of the manuscript. HK: conceptualization, analyzing the data, writing the original draft, and revision and editing of the manuscript. All authors contributed to the article and approved the submitted version.

## Funding

We acknowledge support from the Open Access Publication Fund of the University of Wuppertal.

## Conflict of Interest

The authors declare that the research was conducted in the absence of any commercial or financial relationships that could be construed as a potential conflict of interest.

## Publisher’s Note

All claims expressed in this article are solely those of the authors and do not necessarily represent those of their affiliated organizations, or those of the publisher, the editors and the reviewers. Any product that may be evaluated in this article, or claim that may be made by its manufacturer, is not guaranteed or endorsed by the publisher.
